# Clinical Epidemiology of Low-Grade and Dedifferentiated Osteosarcoma in Norway during 1975 and 2009

**DOI:** 10.1155/2015/917679

**Published:** 2015-08-30

**Authors:** Kjetil Berner, Tom Børge Johannesen, Øyvind S. Bruland

**Affiliations:** ^1^Department of Oncology, Oslo University Hospital, Norwegian Radium Hospital, 0424 Oslo, Norway; ^2^The Norwegian Cancer Registry, 0304 Oslo, Norway; ^3^Institute of Clinical Medicine, University of Oslo, 0318 Oslo, Norway

## Abstract

*Purpose*. To describe epidemiological, clinical characteristics and treatment outcomes of low-grade osteosarcoma (LGOS), including dedifferentiated osteosarcoma (DLGOS). *Method*. We analysed a nationwide cohort comprised of patients with histologically verified LGOS and DLGOS between 1975 and 2009, based on registry sources supplemented with clinical records from hospitals involved in sarcoma management. *Results*. Fifty-four patients were identified, 12 of whom had DLGOS. The annual incidence for all patients was 0.3 per million, with the peak incidence in the third decade of the life. Fifteen patients experienced local relapses during follow-up and ten developed metastatic diseases, including three at primary diagnosis. Patients with DLGOS dominated the metastatic relapse group. The five-year sarcoma-specific survival rate was 91%, with no documented improvement over time. Free margin following surgical resection of the primary tumour had a positive impact on survival. As expected, both local relapse and metastasis during follow-up were associated with an unfavourable outcome. Radiotherapy predicted poor survival due to the selection of high-risk patients in need of such treatment. Neither higher age nor axial tumour localisation was adverse prognostic factors. *Conclusion*. LGOS has an excellent prognosis when surgically resected with a free margin; however, LGOS has the potential to dedifferentiate and metastasize with a poor outcome.

## 1. Introduction

Most osteosarcomas (OS) are high-grade lesions, while low-grade OSs (LGOS) are rare and include parosteal osteosarcoma (POS) and central LGOS (LGCOS) [1]. POS was first described in 1951 [2] and is characterised as a slow growing, low-grade malignancy arising from periosteal tissue directly adjacent to the cortex [1, 2]. LGCOS was first reported in 1977 [3] as a distinct entity of well-differentiated, intramedullary LGOS [1, 3, 4]. POS accounts for approximately 4% of all OS [5, 6] and LGCOS accounts for less than 2% [4, 7].

Most patients in the cohort were young adults with a peak incidence in the third decade of life in both subgroups of LGOSs [5–8]. There is no significant gender difference in the incidence of LGOS [6–9]. The majority of such primary tumours are located in the long bones, most often in the distal femur and proximal tibia, while flat bones are less likely to be affected [1, 4, 8].

In general, both subgroups of LGOSs have excellent prognoses when surgically resected with a wide margin [4, 7, 9]. However, LGOS has the potential to dedifferentiate to high-grade malignant lesions (DLGOS) upon recurrence [5–9]. Some LGOS will also show areas of high-grade malignancy already at primary diagnosis [1, 10]. Adjuvant chemotherapy is recommended in such circumstances [1, 8], due to the increased risk of subsequent metastasis [1, 8, 11].

The purpose of this study is to describe the epidemiological and clinical characteristics related to treatment outcomes in all LGOS and DLGOS from an unselected Norwegian population of OS patients between 1975 and 2009 [12]. To our knowledge, none of the previous nationwide studies [12–16] have specifically addressed this topic.

## 2. Material and Methods

### 2.1. Patient Cohort

Fifty-four cases of LGOS and DLGOS were identified based on histological reports from a population of 702 patients with OS and spindle cell non-OS in Norway between 1975 and 2009 [12, 17]. One hundred and thirty cases were retrieved from files and reexamined due to somewhat questionable pathological reports [12]; of these, 26 are included in the present study. Variables relevant to this study were retrospectively validated based on multiple and partly overlapping data and registry sources supplemented with clinical records from hospitals involved in sarcoma management. The DLGOS subgroup included all patients with dedifferentiated lesions, verified either at primary diagnosis or during follow-up. Malignancy grade was dichotomised between low-grade (grades I-II) and high-grade (grades III-IV) tumours [18]. Two patients had previously been reported with LGOS [12] but received chemotherapy due to small areas with documented grade III malignancy. Consequently, these cases are classified as “DLGOS at diagnosis” in the present study.

### 2.2. Clinicopathological Variables

We defined metastasis that occurred within six weeks of primary diagnosis as* primary metastatic disease* [12]. Information regarding metastasis or local recurrence was based on radiographic images and/or biopsy or fine needle aspiration cytology.* Tumour size* was defined as the maximum length of the tumour in cm and* duration of symptoms* referred to the interval in months between first symptom and time of biopsy [19]. The normal ranges for* serum alkaline phosphatase* (ALP) and* serum lactate dehydrogenase* (LDH) were measured in international units at the time of diagnosis [19].

### 2.3. Treatment Variables

*Surgery.* We dichotomised between amputation and other surgeries. The best local surgical margins were classified as free or positive margins. The former implied surgical removal of the primary tumour with wide or marginal margins (adequate surgery) [20] as defined by the surgeon and pathologist, while an intralesional margin and residual macroscopic tumour were categorized as positive margins. Patients with metastatic disease at the time of diagnosis must have achieved complete surgical remission for both their primary tumour and metastasis in order to be classified as having received adequate surgery. All patients treated with curettage were assumed to have a positive margin.

*Chemotherapy.* Adequate chemotherapy was defined as at least six courses of chemotherapy containing a minimum of two of the following drugs: high-dose methotrexate (at least 8 g/m^2^), doxorubicin, cisplatin, or ifosfamide [19]. These four drugs are the most commonly used chemotherapy drugs worldwide [21, 22].

*Radiotherapy*. A curative treatment intent was defined as fractionated radiotherapy following surgery, for either the treatment of a primary tumour or a local recurrence, otherwise considered as palliative treatment.

### 2.4. Statistical Analyses

A survival analysis using Kaplan-Meier estimates and a log-rank test were used to analyse sarcoma-specific survival (SSS) and event-free survival (EFS). For comparison, we also present SSS for all Norwegian high-grade OS patients between 1975 and 2009 [12, 17]. Overall survival was not used in these analyses since only about half of all deaths in the cohort were due to OS. Sarcoma-specific death or treatment-related deaths were the endpoints of SSS. The endpoints of EFS were date of first metastasis, local recurrence, or SSS, whichever occurred first. Patients with primary metastatic disease were not included in the EFS analysis. Follow-up was completed in July, 2013. Updated registries were used to prevent bias due to nonidentical follow-up of patients with few or frequent appointments [12]. The statistical analyses were conducted using SPSS version 22 (SPSS, Inc., Chicago, IL) and Stata version 13.1 (Stata Corporation, College Station, TX).

## 3. Results

### 3.1. Incidence

**Figure 1:**
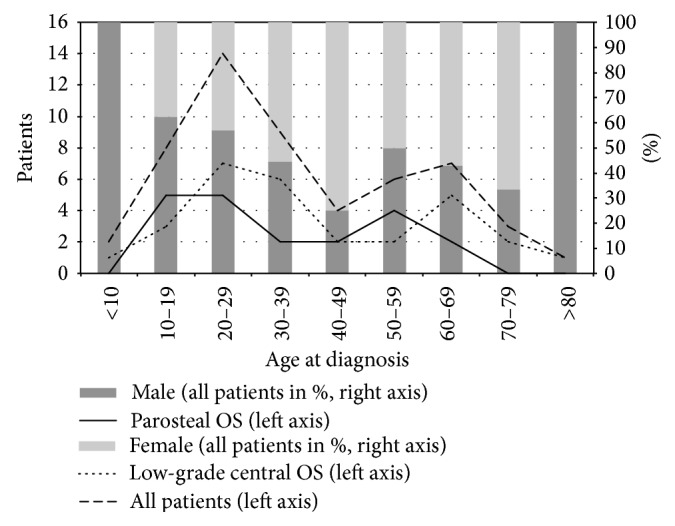
Gender (bar chart, right axis) and age at diagnosis (line diagram, left axis) of 54 patients with low-grade or dedifferentiated osteosarcoma (OS), including separate line diagrams for parosteal and low-grade central OS.

Fifty-four patients were diagnosed with LGOS or DLGOS between 1975 and 2009. These patients represent 11% of all OS during this period [12, 17]. The average annual incidence of 0.3 per million was bimodally distributed by age, with the dominant peak occurring in the patients' twenties (Figure 1). We report no significant gender differentiation of all patients in the cohort (Table 1 and Figure 1).

### 3.2. Clinicopathological Data

The LGCOS group was comprised of 29 patients, making it the largest subgroup in the present cohort (Tables 1 and 2). Interestingly, only 12 of these cases (41%) had tumours located in long bones, while 10 cases (34%) had tumours in the mandible or maxilla (Table 2). Jaw OS accounted for 7% of all skeletal OS, with a LGCOS to high-grade ratio of 32% [12]. With one exception, all 20 cases of POS were located in long bones (Table 2). Four of the remaining five patients were classified as secondary LGOS (Table 1), with two cases arising from previous fibrous dysplasia, one case from a previous giant-cell tumour, and one due to previous radiotherapy. The fifth case of LGOS was located in the left breast of a young female [17, 23].

Twelve patients had DLGOS (Table 1), including six patients with high-grade POS at primary diagnosis. In addition, two cases of POS and four cases of LGCOS showed transformation to high-grade malignancy at the time of local recurrence, that is, five patients at time of first local relapse and the sixth one at time of a third local recurrence. These six DLGOS patients developed transformation to high-grade malignancy between 1 and 21 years after primary diagnosis (Table 3). Four of these six cases had previously undergone surgery with an intralesional surgical margin.

Median tumour size among DLGOSs was 11 cm and nearly twice as large as those of the rest of the cohort (Table 1). By contrast, approximately an equal duration of symptoms before biopsy was seen in both LGOS and DLGOS, that is, half a year in median length (Table 1). Six patients had symptoms for more than five years before an OS diagnosis was documented, due to very slow tumour growth. This explains the 13-month discrepancy between median and mean value for these patients (Table 1). About one-third of all patients had elevated ALP at diagnosis, in contrast to 20% with increased levels of LDH. Two patients had a pathologic fracture at time of diagnosis but only one had this in the weight-bearing lower extremity skeleton.

### 3.3. Local Recurrence and Metastases

Out of the 15 patients who experienced local recurrence, six revealed a high-grade morphology during follow-up (Table 3). Two patients with dedifferentiated POS and one with LGCOS had metastatic disease at time of primary diagnosis. In addition, seven patients developed metastases during follow-up. The patients with DLGOS dominated the group with metastatic relapse (Table 3); five of these eight DLGOS patients had previously experienced a local recurrence.

### 3.4. Treatment Modalities

Table 4 outlines the extent of treatment administered to LGOS and DLGOS patients and is discussed further in the following section.

*Surgery.* All 54 patients underwent at least one operation (Table 4). Only five patients were treated with amputation, including three whose amputations were part of their primary treatment. Information regarding surgical margins after resection of the primary tumour was available in 52 cases, including all cases of DLGOS (Table 4). The remaining two patients in the cohort both experienced local relapses during follow-up but did not develop metastasis.

Seven of the 43 patients that obtained* free surgical margins* after resection of the primary tumour (Table 4) experienced local recurrence during follow-up: four with LGOS and three with DLGOS. Furthermore, five patients with a similar margin status were diagnosed with metastases, including one in a primary metastatic setting. Only one of these five patients developed metastasis with a low-grade malignancy. The remaining nine patients in the cohort did not achieve adequate surgery due to* positive margins*, including four DLGOS patients (Table 4). Three of the four DLGOS patients developed local recurrence and subsequent metastases during follow-up, while the last patient had primary metastatic disease. In addition, three of the five LGOS patients with positive surgical margins experienced local relapse, while another underwent palliative surgery for a pathological humerus fracture due to primary metastatic disease.

*Chemotherapy.* Adjuvant chemotherapy was, as expected, mainly reserved for patients with high-grade malignant lesions (Table 4). Only a 35-year-old man with LGOS received adequate chemotherapy, that is, after a second operation with decompression of the medulla due to a local relapse in the first lumbar vertebra [24].

*Radiotherapy.* Six patients received postoperative radiotherapy with curative intent (Table 4), including one case in which radiotherapy was used to treat the tumour in the appendicular skeleton. Fractionated radiotherapy was given due to marginal surgical margins in two cases and intralesional surgical margins in the remaining four cases. No signs of recurrent disease were later seen in the first two cases, whereas the other patients succumbed to their disease.

### 3.5. Cause of Death

Eight patients died of OS due to metastatic disease: two with LGOS and six with DLGOS. Another three LGOS patients died of OS due to local recurrence; in one case, there was a primary tumour in the mandible, and the other two cases developed tumours in the columna vertebralis. An additional 10 patients in the cohort died of other causes, which included other cancers (four cases), epilepsy (one case), infection (one case), gastric perforation (one case), heart disease (one case), “sudden death” (one case), and suicide (one case).

### 3.6. Survival Analyses

**Figure 2:**
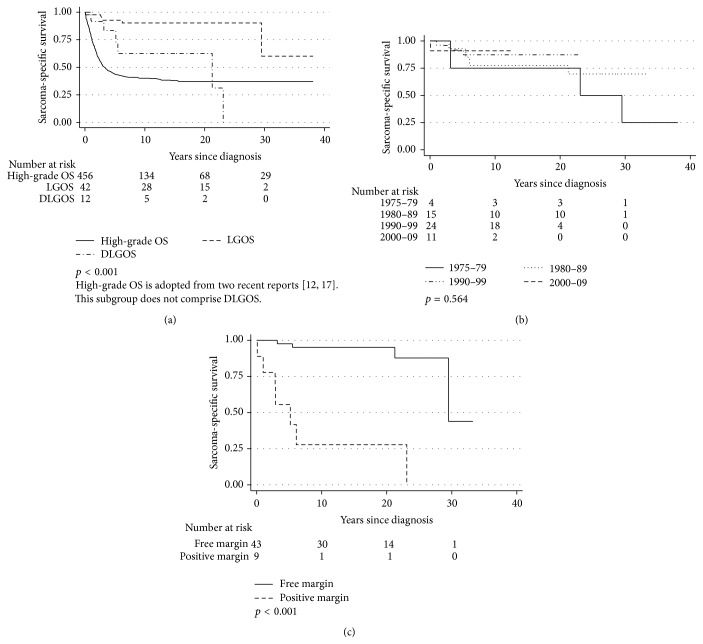
Sarcoma-specific survival of low-grade (LGOS) and dedifferentiated osteosarcoma (DLGOS) versus (a) high-grade OS, (b) dependent on time of diagnosis, 1975–2009, and (c) by best local surgical margin.

Patients with LGOS experienced an improved survival rate compared to both DLGOS and the high-grade OS cohorts (Figure 2(a)) [12, 17]. Five-year SSS was 91% for all patients in the present study and ten-year SSS was 85%. We found no improvement over time for all patients in the study, based on 10-year intervals (Figure 2(b)) and a cut-off in 1990 (*p* = 0.31, data not shown).

Table 5 presents the results of univariate analyses for five-year SSS and EFS according to a variety of characteristics. Gender (*p* = 0.34/0.52), tumour size (*p* = 0.94/0.61), symptom length (*p* = 0.40/0.08), ALP (*p* = 0.49/0.47), and LDH (*p* = 0.84/0.62) were not included in Table 5. Free margin following surgical resection of the primary tumour had a significant positive impact on survival for all patients (Figure 2(c) and Table 5), while local relapse or metastasis during follow-up was associated with a poor outcome (Table 5). Nevertheless, four patients died of metastases from OS despite a free surgical margin of the primary tumour, including three from the DLGOS subgroup. Radiotherapy predicted inferior SSS while chemotherapy had no significant impact on outcome (Table 5). Neither higher age nor axial tumour localisation was adverse prognostic factors.

## 4. Discussion

The patient material studied here is based on a nationwide cohort comprised of all Norwegian LGOS and DLGOS patients within a timeframe of 3-4 decades [12, 17]. To our knowledge, no previous nationwide study has specifically addressed topics related to clinical epidemiology and treatment results for these OS entities, except for separate incidence calculations [12, 15]. LGOS accounts for more than 10% of all OS in both Norway and Finland [12, 15], which is higher than usually reported [4–7]. Our result may be due to a broader patient base than normally reported, both as a consequence of the population-based nationwide approach, but also since we have included, for example, jaw OS, secondary OS, and extraskeletal OS in the present cohort. Since the diagnosis of LGOS in general is challenging, the lack of a systematic histological and radiological reevaluation of all cases in the gross study material analysed (see below) might also contribute to our findings.

The anatomical sites of POS in the present cohort (Table 2) were consistent with those in previous publications [1, 6, 8]. In contrast, the anatomical distribution of LGCOS, with the jaw as the single most frequent site, differs substantially from that of previously published studies in which long bones were the most commonly affected [3, 4]. This discrepancy may be due to chance, partly as a result of the relatively small sample size. For example, in the present cohort, about one-third of all jaw OSs [12] were LGOS, as compared to between 6% and 42% in previous studies [25–27]. Jaw OS accounted for less than 7% of all OSs in Norway [12], which is consistent with the findings from the Mayo Clinic [28].

The wide range in average duration of symptoms prior to diagnosis seemed reasonable due to the indolent tumour biology in most of these cases. We confirmed that patients may experience a slow growing tumour for years [6]. Such tumour growth may also explain why patients with low-grade histology had smaller tumour size at diagnosis (Table 1) than patients with high-grade OS [19].

It has been previously reported that up to 43% of all POS may contain areas of high-grade morphology [6, 29, 30] and develop de novo from low-grade lesions, either at diagnosis or during subsequent relapse. We confirmed these results. In addition, LGCOS has the potential to dedifferentiate [7–9]; four of our cases contained components with high-grade histology at the time of recurrence. As expected, DLGOS had a worse prognosis than LGOS due to the increased risk of metastasis, which is also confirmed in the literature [1, 6, 10, 31].

Overall, the prognosis for all patients in the present study was excellent following surgical removal with a free margin, which is consistent with the literature [6, 7, 10]. Five-year SSS for all patients in the cohort was 91%, with no improvement over time. Chemotherapy and radiotherapy are not routinely required for patients with LGOS [1, 7, 31], as confirmed in this report. Radiotherapy as a treatment modality predicted inferior SSS in the present analysis, probably due to the selection of high-risk patients in need of such treatment. OS is also known to be relatively resistant to radiotherapy [22, 32]. Neither higher age nor axial tumour localisation was adverse prognostic factors in the present study (Table 5), in contrast to the corresponding subgroups of high-grade OSs, which had a poor prognosis [19, 22, 33].

The strength of the present study, in our opinion, is the reliability of the database, which is validated by multiple and partially overlapping data and registry sources. As expected, we have not obtained complete clinical information for all patients in the present study. Nor has it been possible to obtain the same degree of detail regarding certain clinical variables with our approach as compared to a single institutional series and/or clinical trials. Furthermore, we cannot rule out that the quality could have been even better with a uniform histological reexamination of all 702 cases in the gross study material analysed, including immunohistochemical analyses as well as a retrospective review of the radiographic images in relevant cases. Nevertheless, a significant disadvantage of such an approach is the potential lack of histological specimens or radiographic images available for reexamination. This might be an even larger problem in nationwide studies than in studies based on, for example, an institutional series. For example, a previous Finnish study experienced a dropout rate of 34% due to missing original histological specimens [14], which is an unavoidable part of studies conducted in such a setting. Hence, we believe the potential disadvantage will exceed the potential gain of such an approach and that our key variables ensure the inclusion of an adequate amount of details in order to expand our knowledge regarding these rare OS entities.

## 5. Conclusion

To our knowledge, this is the first nationwide study addressing the clinicopathological features of LGOS and DLGOS. We confirmed that LGOS has an excellent prognosis when surgically resected with a free margin. LGOS also has the potential to dedifferentiate and metastasize with a poor outcome.

## Figures and Tables

**Table 1:** Clinicopathological variables in 54 low-grade and dedifferentiated osteosarcoma (OS) patients.

	Low-grade OS^a^ (%)	Dedifferentiated OS (%)	All patients^b^ (%)
All patients	42	12	54
Gender			
Male	23 (55)	5 (42)	28 (52)
Female	19 (45)	7 (58)	26 (48)
Histological subgroups			
Parosteal OS	12 (29)	8 (67)	20 (37)
Low-grade central OS	25 (60)	4 (33)	29 (54)
Secondary low-grade OS	4 (10)		4 (7)
Extraskeletal low-grade OS	1 (2)		1 (2)
Tumour size			
≤6 cm	20 (67)	2 (25)	22 (58)
>6 cm	10 (33)	6 (75)	16 (42)
Median/mean size in cm	6/6	11/12	6/7
Range in cm	1–15	5–30	1–30
Symptom length			
≤6 months	16 (48)	5 (56)	21 (50)
>6 months	17 (52)	4 (44)	21 (50)
Median/mean length in months	7/18	6/27	7/20
Range in months	1–108	4–120	1–120

^a^No dedifferentiation at diagnosis or during follow-up. ^b^Missing values equal the difference between the summarized number from each subgroup in the fourth column and the total number of patients in the study.

**Table 2:** Anatomical distribution of 54 patients with low-grade or dedifferentiated osteosarcoma (OS) combined. Separated between parosteal OS (POS), low-grade central OS (LGCOS), and other subgroups.

	POS (%)	LGCOS (%)	Other (%)^a^	All patients (%)
Humerus	4 (20)	1 (3)		5 (9)
Femur	11 (55)	5 (17)		16 (30)
Tibia	4 (20)	5 (17)	1 (20)	10 (19)
Fibula		1 (3)		1 (2)
Mandible, maxilla		10 (34)	3 (60)	13 (24)
Costa, scapula, clavicle		3 (10)		3 (6)
Columna vertebralis		1 (3)		1 (2)
Pelvis, sacrum	1 (5)	2 (7)		3 (6)
Other		1 (3)	1 (20)	2 (4)
Total	20 (100)	29 (100)	5 (100)	54 (100)

^a^Four cases of secondary low-grade OS and one case of extraskeletal low-grade OS (in the breast).

**Table 3:** Patients with local recurrence and/or metastasis at diagnosis or during follow-up among 54 low-grade or dedifferentiated osteosarcoma (OS) patients.

	Low-grade OS (%)	Dedifferentiated OS (%)	All patients (%)
Local recurrence			
No	33 (79)	6 (50)	39 (72)
Yes^a^	9 (21)	6 (50)	15 (28)
Median/mean in months	27/31	26/94	27/57
Range in months	7–62	15–255	7–255
Metastasis^b^			
No	40 (95)	4 (33)	44 (81)
Yes^c^	2 (5)	8 (67)^d^	10 (19)
Median/mean in months	59/59	33/78	33/74
Range in months	0–117	0–263	0–263

^a^Five with parosteal OS (POS), nine with low-grade central OS (LGCOS), and one with secondary low-grade OS. None of the six patients with high-grade malignancy at time of diagnosis experienced local recurrence during follow-up. ^b^Metastasis at diagnosis or during follow-up. Three patients had primary metastatic disease: one with low-grade OS and two with dedifferentiated OS. ^c^Five with POS and five with LGCOS. ^d^Five patients with metastatic relapse during follow-up had previously experienced a local recurrence.

**Table 4:** Summary of treatment in 54 patients with low-grade or dedifferentiated osteosarcoma (OS).

	Low-grade OS (%)	Dedifferentiated OS (%)	All patients (%)
Surgery	42	12	54
Amputation	3 (7)	2 (17)	5 (9)
Other	39 (93)	10 (83)	49 (91)
Surgical margins^a^	40	12	52
Free margin	35 (87)	8 (67)	43 (83)
Positive margin	5 (13)	4 (33)	9 (17)
Chemotherapy	3	8	11
Adequate chemotherapy	2 (67)	7 (88)	9 (82)
At primary diagnosis	1 (33)^c^	5 (63)	6 (55)
Treatment of local relapse	1 (33)	2 (25)	3 (27)
Not adequate chemotherapy	1 (33)	1 (13)	2 (18)
Radiotherapy^b^	5	5	10
Curative treatment intention	4 (80)	2 (40)	6 (60)
Palliative treatment intention	1 (20)	3 (60)	4 (40)

^a^Surgical margins after last resection of primary tumour during primary treatment or later relapses. Two uncertain cases were not included. ^b^Radiotherapy during primary treatment or later relapses. One patient that underwent radiotherapy with curative intent received later radiotherapy in a palliative setting. ^c^The chemotherapy was terminated after an internal hemipelvectomy, since the operation specimen verified a LGOS. The patient was subsequently followed for ten years after primary surgery with no signs of recurrent disease.

**Table 5:** Univariate Kaplan-Meier analyses of five-year sarcoma-specific survival and event-free survival according to different characteristics of 54 patients with low-grade or dedifferentiated osteosarcoma (OS).

	Sarcoma-specific survival			Event-free survival		
	Patients (%)	5 years in % (95% CI^a^ in %)	*p* ^b^	Patients (%)	5 years in % (95% CI^a^ in %)	*p* ^b^
Primary site of tumour			0.287			0.844
Extremity	33 (61)	91 (74–97)		30 (59)	80 (61–91)	
Axial	21 (39)	90 (74–96)		21 (41)	65 (41–82)	
Age			0.158			0.063
≤40 years	33 (61)	94 (77–98)		32 (63)	81 (62–91)	
>40 years	21 (39)	86 (62–95)		19 (37)	63 (37–80)	
Malignancy grade			<0.001			0.004
Low-grade	42 (78)	93 (79–98)		41 (80)	80 (64–90)	
Dedifferentiated	12 (22)	83 (48–96)		10 (20)	50 (18–75)	
Subgroup of OS			0.738			0.868
Parosteal OS	20 (37)	90 (66–97)		18 (35)	72 (46–87)	
Low-grade central OS	29 (54)	93 (75–98)		28 (55)	75 (55–87)	
Other	5 (9)	75 (13–96)		5 (10)	75 (13–96)	
Local recurrence			0.010			<0.001
No	40 (74)	92 (78–97)		37 (73)	97 (82–97)	
Yes	14 (26)	86 (54–96)		14 (27)	20 (5–42)	
Metastases at diagnosis or during follow-up			<0.001			0.001
No	44 (81)	95 (83–99)		44 (86)	79 (64–88)	
Yes	10 (19)	70 (33–89)		7 (14)	57 (17–84)	
Surgery^c^			<0.001			<0.001
Free margin	43 (83)	98 (83–100)		42 (86)	83 (52–95)	
Positive margin	9 (17)	56 (20–81)		7 (14)	29 (4–62)	
Adequate chemotherapy			0.091			0.447
No	45 (83)	93 (80–98)		44 (86)	74 (58–85)	
Yes	9 (17)	78 (36–94)		7 (14)	71 (26–92)	
Radiotherapy^d^			0.003			0.106
No	48 (89)	94 (82–98)		45 (88)	77 (62–87)	
Yes	6 (11)	67 (20–90)		6 (12)	50 (11–80)	

^a^Confidence interval. ^b^Log-rank test. ^c^Surgical margins after resection of primary tumour, last surgery performed. ^d^Curative treatment intent.
